# Artificial intelligence for optimization of immunotherapy: current applications and transformative potential

**DOI:** 10.3389/fimmu.2026.1777580

**Published:** 2026-05-29

**Authors:** Ali Tarhini, Palak Dave, Shari Pilon-Thomas, Issam El Naqa

**Affiliations:** 1 Machine Learning Department, H. Lee Moffitt Cancer Center and Research Institute, Tampa, FL, United States; 2 Immunology Department, H. Lee Moffitt Cancer Center and Research Institute, Tampa, FL, United States

**Keywords:** agentic AI, artificial intelligence, cellular therapy, foundation models, generative AI, immune checkpoint inhibitors, immunotherapy, multi-modal data integration

## Abstract

Artificial intelligence (AI) is a transformative technology that has captivated the medical world with its potential to optimize cancer treatment and enhance precision oncology. In cancer diagnosis and treatment, various AI technologies have already provided high-level data examination and analytics that preceding innovations were not capable of. Cancer immunotherapy is a treatment that seeks to boost the immune system to recognize and eradicate tumors. It is a field that is constantly evolving, serving as a fertile environment where AI technologies can accelerate discovery and personalize its regimens. In recent years, AI has played an increased role in the optimization of immunotherapy delivery and drug development. Traditional machine learning and its subfield of deep learning algorithms have already impacted response prediction and related tasks, such as patient stratification for immune checkpoint blockade treatment and identifying potent T-cells in the laboratory to develop effective cellular therapies. Additionally, recently developed technologies such as generative AI (gen AI) and foundation models have expanded upon traditional AI algorithms with new applications such as treatment plan generation and adverse event prediction. As innovations such as agentic AI and the model context protocol (MCP) become increasingly available, efficiency and success in immunotherapy development and delivery could further improve. That said, some challenges must be overcome for AI to reach its full potential in immunotherapy. These include concerns related to data quality control, patient safety, and addressing ethical dilemmas. In this article, we briefly review available state-of-the-art AI technologies for immunotherapy and highlight their capabilities. Then, we examine the current AI applications in immunotherapy including cell therapies, checkpoint inhibitors, and cancer vaccines, covering a diverse array of technologies over a wide range of applications. We analyze the datasets used, performance metrics, and downstream tasks, and highlight existing limitations. Subsequently, we discuss some of the obstacles that have prevented AI from routine clinical adoption. Finally, we envision the future of AI in immunotherapy that may include a framework involving an orchestration of multiple specialized AI agents with a human in the loop.

## Introduction

Artificial intelligence (AI) and its continuously evolving abilities have great potential to optimize cancer diagnosis and treatment (1, 2). In recent years, AI has emerged with a plethora of applications in various oncological fields, including the emerging field of immunotherapy (3, 4). Essentially, AI refers to the branch of computer science in which computer systems or machines can carry out tasks that usually require human-level intelligence (5). Machine learning (ML) is a subdivision of AI involving identifying patterns and making predictions from datasets; this occurs without direct programming (5). ML embodies a large portion of current medical AI applications. Many current ML processes are greatly driven by artificial neural networks (ANNs) and their variants. A neural network with a large number of hidden layers is often denoted as a deep neural network (DNN) (6, 7). The training of these networks is known as deep learning (DL), which is a subfield of ML used for datasets that are more extensive and vaster than those with traditional ML algorithms, and can operate directly on the input data without the need for manually crafted features.

Many of the technologies mentioned above are heavily involved in *predictive AI*, which makes predictions based on provided data and has been the focus of a recent review for applications in immunotherapy (4). In contrast, *generative AI* (gen AI) technologies are able to generate new content, whether that be text, images, or art, from given inputs (1, 7). With predictive AI already making an impact in the immunotherapy space, gen AI has also begun to spark discussion about its potential use. Combining predictive and gen AI capabilities can personalize and optimize immunotherapy on both the patient’s and tumor’s levels.

In the early years, AI applications in medicine largely fell under *narrow AI*, which can only perform specific tasks and concentrate on predefined objectives (8). Recently, however, *general AI* has been increasingly utilized; it is a type of AI that can perform a wide range of different tasks (1). Large language models (LLMs), for instance, employ generative and general AI to process and generate human-like language. LLMs and many other technologies are examples of foundation models, large-scale general AI systems that are trained on vast amounts of complex data and can be adjusted for a diverse array of downstream tasks (9). Self-supervised learning (SSL), which increases data efficiency through the direct attaining of guidance from the provided data, is a common way to train foundation models that removes the need to rely on extensive labeling (i.e., supervised learning) (1, 9). Additionally, foundation models can be classified beyond general AI; if the model makes predictions based on provided data, it is considered a general predictive model, while if it generates new content, it is classified as a general generative model.

A technology that is recently emerging but highly promising is *agentic AI*, or the utilization of AI agents. Essentially, AI agents are programs that can interact with their individual circumstances, make decisions, and carry out various tasks in a uniquely autonomous manner, without human input (10). AI agents utilize the capabilities of foundation models to reason and to digest intricate tasks down into simpler ones while also incorporating information from surrounding environments and circumstances. Additionally, the model context protocol (MCP) has been recently developed to drive communication in these AI agent/foundation model systems. In a multi-agent system with a foundation model, MCP can facilitate proper resourcing and communication to increase task efficiency and performance, looking to provide a baseline for how AI systems connect to data sources (11). Agentic AI and MCP represent the next generation of impactful AI technologies in medicine, expanding past the already innovative foundation models (11).

In cancer immunotherapy, the treatment can be largely divided into checkpoint inhibitors, cellular therapy, and cancer vaccines, where most of the AI research has been applied. Immunotherapy drugs such as immune checkpoint inhibitors (ICIs) target immune checkpoint proteins such as PD-1, LAG3, and CTLA-4 to ensure that T-cells remain active and can effectively destroy cancer cells (12). In cellular therapy, a patient’s own immune cells, usually T-cells, are expanded or modified to fight cancer. In CAR T-cell therapy, a patient’s T-cells are genetically engineered to express a chimeric antigen receptor (CAR), which binds to cancer cells. This increases tumor specificity and helps to decrease the likelihood that healthy cells are attacked (13). On the other hand, tumor-infiltrating lymphocyte (TIL) therapy is another type of cell therapy that involves the surgical resection of the tumor and the *ex vivo* expansion of T-cells contained within the tumor. These cellular therapies have provided great optimism as an effective cancer treatment. While CAR T-cell therapy and TIL therapy are the only currently FDA-approved cellular therapies, there are a number of other treatments that are in development, including T-cell receptor (TCR) therapy and the use of natural killer (NK) cells (14). Cancer vaccines have also been developed, with experimental therapeutic vaccines that include antigen-presenting cells stimulating tumor-reactive T-cells to induce powerful, targeted immune responses (13). Integrating specialized AI agents into treatment development and delivery can greatly enhance effectiveness.

**Figure 1:**
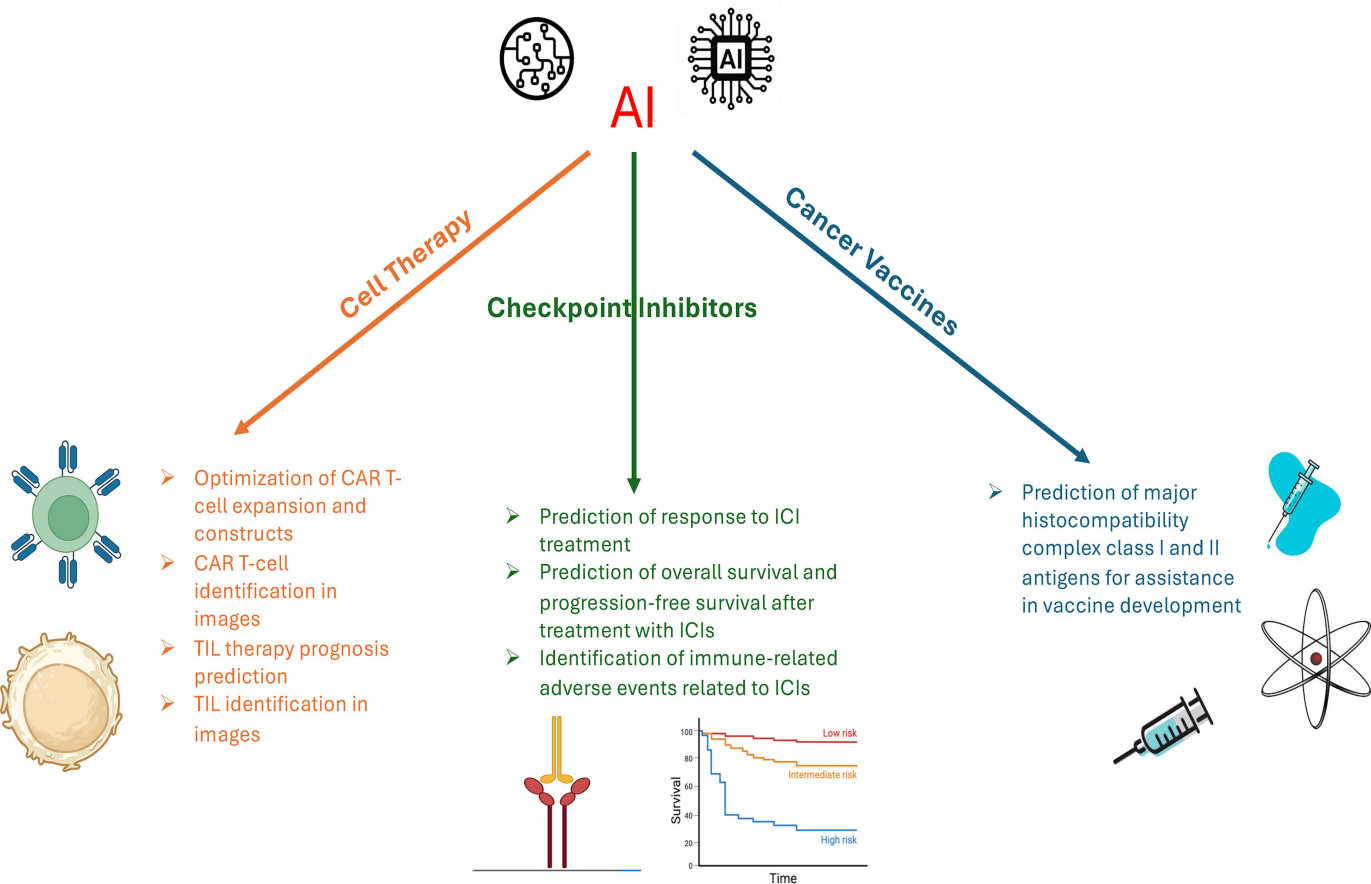
Potential AI applications for different types of immunotherapies. The versatility of AI allows it to carry out a diverse range of downstream tasks spanning multiple immunotherapy regimens. From optimizing CAR constructs in CAR T-cell therapy, to predicting response to ICIs, to assisting in the development of cancer vaccines, and other important tasks, there are a wide variety of ways to utilize AI for the enhancement of immunotherapy.

AI has shown immense promise to transform the science and application of immunotherapy through current applications and future potential. In this article, we review growing AI applications in immunotherapy, particularly in checkpoint inhibitors, cell therapy, and vaccines, where most of the AI research has been applied. The role of predictive AI, as well as the emerging role of gen AI in immunotherapy, is emphasized. This article also discusses the future role of AI in immunotherapy, viewing current and possible future applications of foundation models and AI agents in the field. Limitations and hindrances that must be overcome for AI to reach universal clinical translation are also discussed. **Figure 1** depicts potential AI applications for different immunotherapy regimens that will be further discussed later in the manuscript.

## Methods

This review article identifies, selects, and synthesizes information from multiple sources (e.g., Pubmed, Google Scholar, arXiv) to depict the current state-of-the-art regarding AI applications in studies of immunotherapy. To select literature for inclusion in this article, a search was first conducted on PubMed using a systematic approach. The initial search included keywords such as “artificial intelligence,” “machine learning,” “cancer,” and “immunotherapy,” and was narrowed down to only include original research (clinical trials, studies, etc.) done in the last 5 years. The search was further specified to only identify articles that discuss the development of original AI tools that have been used to optimize the study and delivery of immunotherapy. Finally, articles were screened to determine if they were suited for discussion in either an ICI, cellular therapy, or cancer vaccine context. The PubMed search string is described in **Supplementary Data Sheet 1**, and is depicted in supplementary **Supplementary Data Sheet 2**, which also presents the inclusion/exclusion criteria applied. Given that AI is relatively new to the immunotherapy space, there may be a lack of abundance with regard to validated AI tools in the optimization of this field. Therefore, to supplement this systematic approach, an additional search was conducted on Google Scholar using a scoping approach, where keywords of “artificial intelligence,” “immunotherapy,” and either “cell therapy”, “ICI” or “cancer vaccine” were utilized to identify any additional cutting-edge tools that may have an important emerging role in immunotherapy. A search of this exact nature was also conducted on arXiv to potentially locate any additional novel preprint articles.

This review covers a wide range of applications spanning the most prevalent types of immunotherapy: checkpoint inhibitors, cell therapies, and cancer vaccines. This article combines current and future impacts that AI can have in order to paint a picture of the promising future of the optimization of immunotherapy, largely driven by AI. **Table 1** summarizes representative examples of AI in immunotherapy, briefly explaining each tool and its downstream task capabilities.

**Table 1:** Summary of representative examples of AI tools in immunotherapy, including classifications and capabilities.

AI example	Immunotherapy relevance	Type of AI	Type of training data	Training data availability	Predictive performance	Downstream tasks and limitations
LORIS (15)	Immune Checkpoint Inhibitors	Shallow Predictive (logistic regression)	Pathologic, Genomic, and Clinical Data	Public	Pan-Cancer AUC of 0.75 (95% CI:0.70-.80) NSCLC AUC of 0.74 (95% CI:0.68-0.80)	Predicts response to ICI treatment; however, since the study is retrospective, validation can be done through prospective studies
SCORPIO (16)	Immune Checkpoint Inhibitors	Shallow Predictive (Ensemble of ridge Cox, SSVM, RSF)	Blood tests and clinical data	Private (Requests are Reviewed)	Median AUC of OS is 0.76. AUC of response is between 0.64 and 0.71	Predicts response to ICI treatment; however, since the study is retrospective, validation can be done through prospective studies
IrAE-GPT (17)	Applicable to All Types of Immunotherapy	Deep Generative Foundation Model (GPT)	Clinical Trial and Electronic Health Record Data	Private (Requests are Reviewed)	Overall Micro-averaged F1: Ranges from 0.46 to 0.48 for patient-level evaluations and 0.50 to 0.57 for note-level evaluations.	Identifies immune-related adverse events; despite this, there are not many comprehensive lists for all adverse effects, which can limit the model’s universality
COMPASS (18)	Immune Checkpoint Inhibitors	Deep Predictive Foundation Model (Transformer)	Bulk RNA-Sequencing Data	Public	Accuracy from 0,62-0.72	Predicts response to ICIs; that said, the model is reliant on bulk RNA sequencing data
MUSK (19)	Immune Checkpoint Inhibitors	Deep Predictive Foundation Model (Multimodal transformer)	Pathology Images and reports	Public	AUC of 0.76 (95% CI: 0.73–0.81)	Predicts response to ICIs; however, its multimodal capabilities would require increased computational power
AIDPATH Research Project (20)	Cell Therapy	Shallow Machine Learning (ANN)	Biomarkers and Clinical Data	Private	NA	Assists in the optimization of CAR T-cell expansion
RCMNet (21)	Cell Therapy	Deep Learning (CNN and transformer)	Microscopy Images	Private	Accuracy of 99.63% on a peripheral blood cell (PBC) dataset and 83.36% CAR-T cell dataset.	Identifies PBC and CAR T-cells with good accuracy
CAR-Toner (22)	Cell Therapy	Deep AI-Driven Platform (AlphaFold, ESM2)	Protein Sequences/Expression	Public	NA	Quantifies positively-charged patches that initiate tonic signaling; however, the tool lacks efficiency
InflaMix (23)	Cell Therapy	Shallow Clustering (GMM)	Cytokines and clinical Data	Private (Requests are Reviewed)	AUC of 0.74 is reported.	Detects end-organ function and inflammation; that said, the study carried out was retrospective and needs prospective work to address biases
TRTPred (24)	Cell Therapy	Shallow Machine Learning (logistic regression)	Single cell RNA/TCR sequencing Data	Public	Wide range of AUCs is reported from 0.55 to 0.93	Predicts tumor-reactive T-cell signatures that indicate TIL therapy success
Lunit SCOPE IO (25)	Cell Therapy	Deep AI-Driven Analyzer (DeepLabV3+)	WSI Pathology Images	Private (Upon Request)	Macro F1 score of 0.71 for TIL detection	Analyzes iTIL/sTIL densities for prognostic predictions; however, the study did not take the subtypes of lymphocytes into account
Tarpon (26)	Cell Therapy	Deep Generative Foundation Model (convolutional variational autoencoder)	TCR/RNA Sequencing Data	Public	NA	Generates TCRs for use in therapy; however, uneven TCR chain distribution in the model’s dataset
OnmiMHC (27)	Cancer Vaccines	Deep Machine Learning (multiple deep learning architectures: CNN, LSTM, CBAM, MLP)	Mass spectrometry Data	Public	AUC of 0.85 for MHC-I and 0.61 for MHC-II.	Predicts antigen peptide presentation via MHC class I and II molecules; however, class II data is underrepresented
Adapted ProtT5 (28)	Cancer Vaccines	Deep Predictive Foundation Model (Fine-tuned ProtT5)	Protein Sequencing Data	Public	AUC of 0.93 is reported.	Predicts MHC class II antigens. Further validation is needed.

SSVM, Support Vector Machines; RSF, Random Forest; GPT, Generative Pre-trained Transformer; ANN, Artificial Neural Network; CNN, Convolutional Neural Network; ESM2, Evolutional Scale Modeling 2; GMM, Gaussian Mixture Model; LSTM, Long Short-Term Memory; CBAM, Convolutional Block Attention Module; MLP, Multi-Layer Perceptron; TCR, T-Cell Receptor; WSI, Whole Slide Image; ICI, Immune Checkpoint Inhibitor; CAR, Chimeric Antigen Receptor; sTIL, stromal Tumor Infiltrating Lymphocyte; iTIL, intratumoral Tumor Infiltrating Lymphocyte; NA, Not Available.

## AI in checkpoint inhibitors

### Predictive AI in checkpoint inhibitors

AI has been primarily utilized for ICI response prediction, as there is a current lack of a reliable, accurate tool for predicting patient response (29–32). As summarized in **Table 1**, LORIS (Logistic regression-based immunotherapy-response score) is an example of a shallow predictive AI model for addressing this issue; it is trained on a dataset of 2,881 ICI-treated and 841 non-ICI treated patients across 18 solid tumor types; the dataset spans over 20 pathologic, genomic, and clinical features (15). The selected model was a six-feature logistic LASSO (least absolute shrinkage and selection operator) regression model that included the following features in descending importance order: tumor mutational burden (TMB), systemic therapy history, blood albumin, blood neutrophil–lymphocyte ratio (NLR), age, and cancer type. The study included a pan-cancer model and a non-small cell lung cancer (NSCLC)-specific model. The pan-cancer LORIS model posted a 15-68% increase in area under the receiver operating curve (AUC) over TMB only (AUC of 0.75 [95% CI:0.70-.80]), whereas the NSCLC-specific LORIS showed a 4-17% AUC increase on the PD-L1 biomarker and a 5-23% increase over the TMB biomarker (AUC of 0.74 [95% CI:0.68-0.80]), both approved by the FDA for this purpose (15). This performance underscores the ability of AI to enhance response prediction over current methods, which is especially important given the need for a tool that can stratify patients based on response (15). Another AI tool with a similar function to LORIS is SCORPIO (Standard Clinical and labOratory featuRes for Prognostication of Immunotherapy Outcomes), a predictive AI tool that specializes in survival prediction and clinical benefit prediction, and is trained on clinical data from 17 cancer types, encompassing 1,628 patients, and externally tested on 4,447 ICI-treated patients in 10 global phase 3 clinical trials (16). SCORPIO was trained as an ML ensemble of three shallow ML algorithms with soft-voting: a statistical survival model (ridge Cox regression), and two survival extension models of support vector machine (SSVM) and random forest (RSF). SCORPIO was also shown to provide significant improvement over PD-L1 and TMB with a median AUC of overall survival (OS) of 0.76 and AUC of response between 0.64 and 0.71. However, both studies used a retrospective design, which means that more prospective validation may be required to ensure the generalizability of these models with head-to-head comparisons.

### Generative and general ai in checkpoint inhibitors and treatment management

LORIS and SCORPIO have shown great promise as narrow predictive AI tools; that said, gen AI and general AI can be used to improve various aspects of immunotherapy beyond this. General AI’s ability to be applied to a wider range of downstream tasks increases the areas in which it can enhance immunotherapy drug treatment. IrAE-GPT, for example, is a general generative AI system leveraging OpenAI GPT deep learning models trained on clinical trial and electronic health record (EHR) data to identify immune-related adverse events (IrAEs), which can stem from drugs such as ICIs (17). IrAE-GPT displayed high performance in a variety of IrAEs, with the leveraged GPT (Generative Pre-trained Transformer) models displaying high F1-scores (0.67-1.0) for lupus flare, ascending flaccid paralysis, hemolytic anemia, etc. (17) However, other IrAEs were subject to weaker performance, with the GPT-4 model yielding F1 scores of 22% for arthritis and 31% for hypothyroidism (17). Its overall micro-averaged F1: ranged from 0.46 to 0.48 for patient-level evaluations and from 0.50 to 0.57 for note-level evaluations. This variation signals significant room for improvement regarding the use of GPT models in IrAE studies, particularly in handling textual causation, where adverse events are only identified in clinical text but also linked to other treatment confounders (17). A similar study was carried out with the open-source LLM OpenOrca, used to identify colitis, pneumonitis, hepatitis, and myocarditis (17, 33).

Predictive foundation models have also shown potential, improving upon traditional methods in ICI response prediction. COMPASS, for example, is a deep learning foundation model trained on bulk RNA-sequencing data from 10,184 tumors spanning 33 cancer types (18). For ICI response prediction, COMPASS improves accuracy by 8.5% and area under the precision-recall curve (AUPRC) by 15.7% over 22 existing models and biomarkers such as PD1 and CTLA4 in 16 distinct clinical cohorts covering 6 ICIs and 7 cancers (18). However, limitations include a reliance on bulk-RNA sequencing data, which fails to provide much cellular and spatial resolution and can conceal relevant signals from rare immune cell populations. To improve this issue, single-cell and spatial transcriptomics could improve the resolution of intercellular interactions and cell-specific immune states (18, 34–36). In addition, predictive foundation models have also been utilized in tertiary lymphoid structure (TLS) identification, as a spatial atlas of over 1000 NSCLC tumor tissues was built and used to train a multimodal foundation model from multiple paired modalities: spatial transcriptomics, immunofluorescence images, hematoxylin-eosin (H&E) stained images, and whole exome sequencing (37).

Pathology foundation models employing general predictive AI have also impacted ICI treatment optimization. MUSK is such a model, utilizing general predictive deep learning AI for ICI response prediction. MUSK (Multimodal transformer with Unified maSKed modeling) is a vision-language foundation model, combining computer vision and natural language processing to digest both images and text (19). It was pretrained on 50 million pathology images from 11,577 patients and one billion pathology-related text tokens. This includes one million pathology image–text pairs for efficiently aligning visual and language features. For response prediction evaluation, a multimodal dataset including pathology reports and associated H&E slides for 118 patients with advanced NSCLC treated with ICIs was collected (19). MUSK was then evaluated for progression-free survival (PFS) and objective response. For predicting response, MUSK displayed an AUC of 0.768, compared to an AUC of 0.606 from tumor PD-L1 expression. Moreover, for predicting PFS, MUSK produced a c-index of 0.705, compared to 0.574 for tumor PD-L1 expression (19). MUSK’s immunotherapy response prediction ability is promising and shows potential for finding an accurate marker for stratification and the identification of patients suitable for immunotherapy, addressing a pressing need in the immunotherapy field (38–41). Similarly, Patho-GPT is another pathology foundation model trained with 13,770 whole slide images (WSIs) from 6589 patients and evaluated for outcome prediction of ICIs in NSCLC (42). The model achieved an AUC of 0.774 in the internal set and an AUC of 0.752 externally.

## AI applications in cell therapy

### Predictive AI in cell therapy

AI is being used increasingly in the science and application of TIL therapy and CAR T-cell therapy, especially in the latter. Various aspects of CAR T-cell therapy, such as expansion, CAR constructs, etc., have incorporated AI to enhance production efficacy. For instance, an AIDPATH (AI-driven, Decentralized Production for Advanced Therapies in the Hospital) research project beginning in 2021 focused on the bioreactor, where CAR T-cells are cultivated. In this project, a digital twin of the bioreactor was developed to formulate predictions that indicate when CAR T-cell expansion should be terminated (20). Additionally, an ML-based method using shallow artificial neural networks (ANN) was developed to quantify CAR T-cell immunological synapse (IS) images, as CAR IS quality has been shown to correspond with antitumor activities (43, 44). Another recent innovation in CAR T-cell therapy is RCMNet (ResNet18 with Convolutional Block Attention Module and Multi-Head Self-Attention), a deep learning model using convolutional neural networks (CNN) and transformer architecture to identify CAR-T-cells in microscopy images of peripheral blood (21). The model is trained on a CAR-T dataset with 500 original microscopy images for leukemia treatment. RCMNet displayed 99.63% accuracy on a peripheral blood cell (PBC) dataset and 83.36% CAR-T cell dataset (21). This drop in performance is attributed to the smaller training sample size, which can create insufficient generalization power (21).

CAR T-cell therapy is an area where intricate innovations are constantly occurring. In fact, CRISPR Cas-9 utilization has been proposed to enhance the therapy. Using CRISPR Cas-9 to disrupt genes that play a role in immune checkpoint pathways can increase CAR-T-cell resistance to exhaustion, enhancing performance, persistence, and anti-tumor responses (45). Moreover, CNNs can be utilized to determine DNA sequences that are best suited for CRISPR Cas-9, allowing it to be better used in CAR T-cell therapy contexts (45). In addition, CAR-Toner is an AI-driven approach used for the quantification of positively charged patches (PCPs) that can trigger tonic signaling, which is pivotal in CAR T-cell therapy: if tonic signaling is insufficient, then CAR-T resistance becomes poor, and if excessive, CAR-T exhaustion can occur (22, 46–48). CAR-Toner is developed by training an AI model on AlphaFold predictions and sequences; the model is then fine-tuned using Evolutional Scale Modeling 2 (ESM2), a transformer-based language model with an attention mechanism to capture interaction patterns between pairs of amino acids in the input sequence, becoming CAR-Toner. The PCP calculator was trained on a dataset comprising of 170,000 protein sequences along with their associated PCP scores from the Protein Data Bank. The ESM2 model is pre-trained on over 60 million protein sequences from the UniProt Reference Clusters (UniRef) database. The approach displayed an *R* value of 0.94, displaying high performance for PCP quantification (22). However, CAR-Toner is limited by a lack of efficiency, with each calculation taking multiple days (22).

Regarding the manufacturing of CAR T-cells, AI has not only been integrated in cell expansion, as discussed earlier, but also in overall development efficiency and its bioinformatics pipeline. Currently, CAR T-cell therapies often depend on manual analytical processes, causing potential delays in treatment delivery and administration (49). To solve this problem, an AI-driven analytical platform has been developed. Essentially, AI-based analytical testing for CAR T was implemented with cartridge-based flow cytometry, impedance-based real-time cell analysis, and cartridge-based cytokine detection (49). Automated versus manual flow cytometry was carried out, and the AI tool was able to locate patterns and relationships that traditional bioinformatics methods may not have recognized (49).

AI’s current role in cell therapy also involves predicting, identifying, and limiting treatment side effects. For example, Bogatu et al. utilized ML and meta-review informed multi-perspective prediction algorithms for the identification and diagnosis of cytokine release syndrome (CRS), a main side effect of CAR T-cell therapy (50). InflaMix (INFLAmmation MIXture Model) is another AI tool impacting this area. The technology uses an unsupervised Gaussian mixture model (GMM) to capture inflammation and end-organ function using clinical data (14 pre-CAR T infusion laboratory and cytokine measures) (23). InflaMix was able to identify patients with increased likelihood for disease relapse and mortality in a consistent and reproduceable manner with a reported AUC of 0.74 (23). That said, InflaMix does not take into account all factors that may contribute to CAR T efficiency, which may include CAR T-cell quality and target antigen selection (23).

One specific complication of CAR T-cell therapy is Immune Effector Cell-Associated Neurotoxicity Syndrome (ICANS). Currently, there is a lack of quantitative diagnostic criteria for this issue that has been completely characterized (51). In order to address this, a study was carried out where all patients with non-Hodgkin Lymphoma or acute lymphoblastic leukemia who underwent CAR T-cell therapy from 2018 to 2024 were analyzed (51). Then, available post-infusion brain MRIs were analyzed by a CNN algorithm to quantify T2 fluid attenuated inversion recovery (FLAIR) volumetrics; linear mixed regression models then analyzed the DL-driven FLAIR. As a result, it was found that patients with ICANS had much greater FLAIR, showing that this DL-based MRI quantification can provide a quantitative biomarker for ICANS (51).

While most AI cell therapy applications pertain to CAR T-cell therapy, the technology has also impacted the more recently FDA-approved TIL therapy. From prognostics using TILs to TIL therapy optimization, AI has displayed versatility in overall impact with TILs. For instance, TRTpred is an AI-driven predictive model benchmarked with an ML framework based on regularized shallow learning logistic regression that predicts tumor-reactive T-cell (TRT) signatures from tumor-reactive T cell receptors (TCRs) single cell sequencing, which are an important indicator of TIL therapy efficacy (24). The model first predicts cell-wise tumor specificity from single-cell RNA sequencing (scRNA-seq) data, which is then inferred on the TCR repertoire. The model can also investigate the immune reserve of tumor-reactive TILs spanning various tumor microenvironments and indications. The model is trained on 235 CD8^+^ clonotypes from 10 metastatic melanoma patients, with cells and clones annotated as tumor-reactive or non-tumor-reactive (24). Essentially, the model helps determine which TILs enter clinical therapy based on promise for successful treatment.

Separate from TIL therapy, the identification of TILs has been utilized to define a patient’s tumor microenvironment. The involvement of AI in this process has helped determine whether patients are strong candidates for immunotherapy or not. For instance, Lunit SCOPE IO is an AI-powered H&E WSI analyzer for assessing intratumoral TIL (iTIL) densities and tumor-related stromal TIL (sTIL) densities (25). The AI method was based on deep learning models using DeepLabV3+ CNN architecture, with a ResNet-34 backbone network (25). H&E WSI from 289 patients were used in the study, which tested the model’s ability to predict prognosis in patients with colon cancer treated with surgery and adjuvant therapy (25). It was found that, using the tool, patients with established recurrences had much lower sTIL densities (630.2/mm (2)) than those with no recurrence (1021.3/mm (2)) (25). The model achieved an intersection over union (IoU) score of 0.82 for segmentation of cancer area, and 0.67 for cancer stroma, and macro F1 score of 0.71 for TIL detection. However, the study did not consider the subtypes of lymphocytes, and future research may be needed to enhance predictive accuracy by incorporating additional relevant features, such as detailed spatial boundary delineation, etc. (25).

As mentioned, pathologists have played an important role in enhancing the TIL identification and analysis for immunotherapy candidacy, with AI applications also growing in this field. In fact, a study was conducted comparing the performance of AI-driven TIL identification to human pathologists. In essence, 60 H&E-stained melanoma tissue sections were examined using both human and AI-based analysis, and intraclass correlation coefficient (ICC) scores were computed (52). The AI algorithm was able to achieve an ICC of over 0.90 for all ML TIL variables, outperforming manual TIL identification and displaying the potential of AI in pathology-based TIL studies (52). Overall, digital WSI analysis in pathology can play a significant role in many immunotherapy-based objectives in the future (53–55).

### Generative and general AI in cell therapy

Gen AI and general AI have made major impacts on oncology in recent years, providing a glimpse into the future as far as their potential. While the previously mentioned examples are promising, they are focused on narrow predictive analytics. Gen AI and general AI technologies, on the other hand, can expand past this to further enhance cell therapy. One example of this is Tarpon, a Gen AI model for the innovative TCR therapy. The AI tool is based on a convolutional variational autoencoder. In terms of data, 342,844 complementarity-determining region (CDR3)αs, 753,755 CDR3βs, and 54,883 Ag-resolved CDR3αβ pairs were compiled. Tarpon can potentially produce TCRs for any sort of biological distribution, like for a certain antigen (26). Tarpon can be utilized to enhance TCR therapy through the specific generation of TCRs that are highly inclined to recognize a specific antigen, ensuring that the predicted TCRs are more likely to work effectively (26). That said, Tarpon may be limited by its discovery dataset, as it contained far fewer CDRα chains than CDRβ chains (26).

LLMs are also impacting cell therapy in innovative ways. For instance, scChat is an LLM based on OpenAI GPT-4o model that can enhance CAR T-cell therapy with high-level analysis and output generation (56). This general generative AI technology is trained on single-cell RNA sequencing data and is capable of validating hypotheses, explaining failed hypotheses, and contextualizing single-cell RNA sequencing analysis (56). In addition, outside of cell therapy, in the identification of TILs for defining the tumor microenvironment, general-purpose pathology foundation models such as Prov-GigaPath have been increasingly utilized. This general predictive foundation model, originally developed for cancer subtyping and mutation prediction, has been subject to ongoing studies where it is evaluated for the identification of TLS in WSIs (57). The model is trained on a large dataset comprising 1.3 billion 256x256 pathology image tiles, covering 31 major tissue types and 30,000 patients (57). Overall, the future of cell therapy holds great promise, with Gen AI and general AI being able to provide analysis and outputs that previous narrow and predictive models are not capable of.

## AI applications in cancer vaccines

Tumor vaccines are a relatively novel advancement and are still largely experimental. Since this approach is less established in cancer immunotherapy, there is still not an abundant amount of AI applications in this area. That said, there have been noteworthy innovations regarding AI in this field as of late, beginning with the use of predictive AI to aid in vaccine development optimization. Jian et al., for example, developed an ML framework named OnmiMHC for antigen peptide presentation prediction by major histocompatibility complex (MHC) class I and II molecules (27). OnmiMHC comprises the following components: (i) a 2D CNN for extracting high-level abstract features from sequences of MHC molecules and peptides, (ii) a Convolutional Neural Network - Bidirectional Long Short-Term Memory (CNN-BiLSTM) for extracting binding sequences of MHC molecules and peptides, (iii) a Convolutional Block Attention Module (CBAM) module for attention on features, and (iv) a multi-layer perceptron (MLP) classifier to predict the binding between peptides and MHC molecules. OnmiMHC is capable of predicting peptide-MHC binding affinities spanning MHC class I and II molecules, providing optimism for a possible framework for developing a tumor vaccine. In fact, the model displays an AUC of 0.854 for an MHC-I predictive task and a score of 0.606 for such a task with MHC-II (27). Despite this promise, there is currently much more MHC-I data than that available for MHC-II, proving to be an obstacle for this technology’s routine clinical use. Nonetheless, OnmiMHC is a promising technology that can advance AI in cancer vaccines.

General AI has also made breakthroughs in this field. Xu and colleagues adapted the ProtT5 model, a general-purpose protein language model (58), to address the lack of investigation into TCR prediction of MHC class II antigens. The ProtT5 model was fine-tuned with a feed-forward neural network structure on MHC class II antigen peptide and TCR datasets. For TCR prediction of MHC class II antigens, ProtT5 attained an AUC of 0.93 and a prediction accuracy of 0.96, displaying promise for the future enhanced identification of relevant tumor antigens and assistance in the development of cancer vaccines (28).

## Discussion

The future of AI in immunotherapy features the innovative progression of gen AI, as well as AI agents and the MCP-facilitated real-time inter-connection between AI host applications and LLM servers. Regarding gen AI, its combined use with predictive AI can uncover novel approaches and methods for optimizing the science and application of cancer immunotherapy. Further expanding on gen AI, predictive foundation models, and their ability to extract features from even smaller datasets, sets them apart from narrow AI models. These large-scale models have made headway in applications such as ICI response prediction, proving to be more effective than existing biomarkers and improving upon existing narrow AI technology. Building upon this further, AI agents can leverage foundation model capabilities while interacting autonomously with their environment to comprehend and adapt at an enhanced rate. Combining this ability with MCP allows for standardization and efficiency in completing end-to-end immunotherapy tasks, from automating manufacturing and optimizing the bioinformatics pipeline to patient stratification.

### Future optimization of cancer immunotherapy with predictive and generative AI

Predictive and gen AI target different aspects of cancer immunotherapy, but when used in conjunction, can cover a wide range of applications and improve upon a great number of aspects of the treatment. In the context of ICIs, when incorporated with predictive AI, gen AI can identify side effects to these drugs, while general predictive AI can be leveraged to predict patient response. Regarding cell therapies, predictive AI can analyze patient information to similarly determine treatment response, and gen AI models like LLMs can transform this analysis into comprehensive, interpretable language and present it to clinicians as a report. Additionally, gen AI can be used to improve and build upon current predictive AI tools. For instance, RCMNet is discussed as a predictive tool for the identification of CAR T-cells in blood. Once these cells are identified, their genetic profiles can be fed into gen AI models to develop tailored treatment plans based on the cells identified in specific patients. Similarly, predictive AI can determine and identify optimal CAR-T constructs, such as extracellular, transmembrane, and intracellular signaling domains, which can possibly be generated by gen AI in the form of design concepts or optimal protein sequences in the future. In TIL therapy, if predictive AI identifies the TILs with the highest chances for treatment success, gen AI could possibly be used in the future to generate these design concepts for researchers, who can utilize this information to expand the optimal cells. NK and TCR therapies can also make future impacts, with Gen AI already proposed as a TCR optimizer with the Tarpon model.

### The future role of agentic AI in cancer immunotherapy

**Figure 2:**
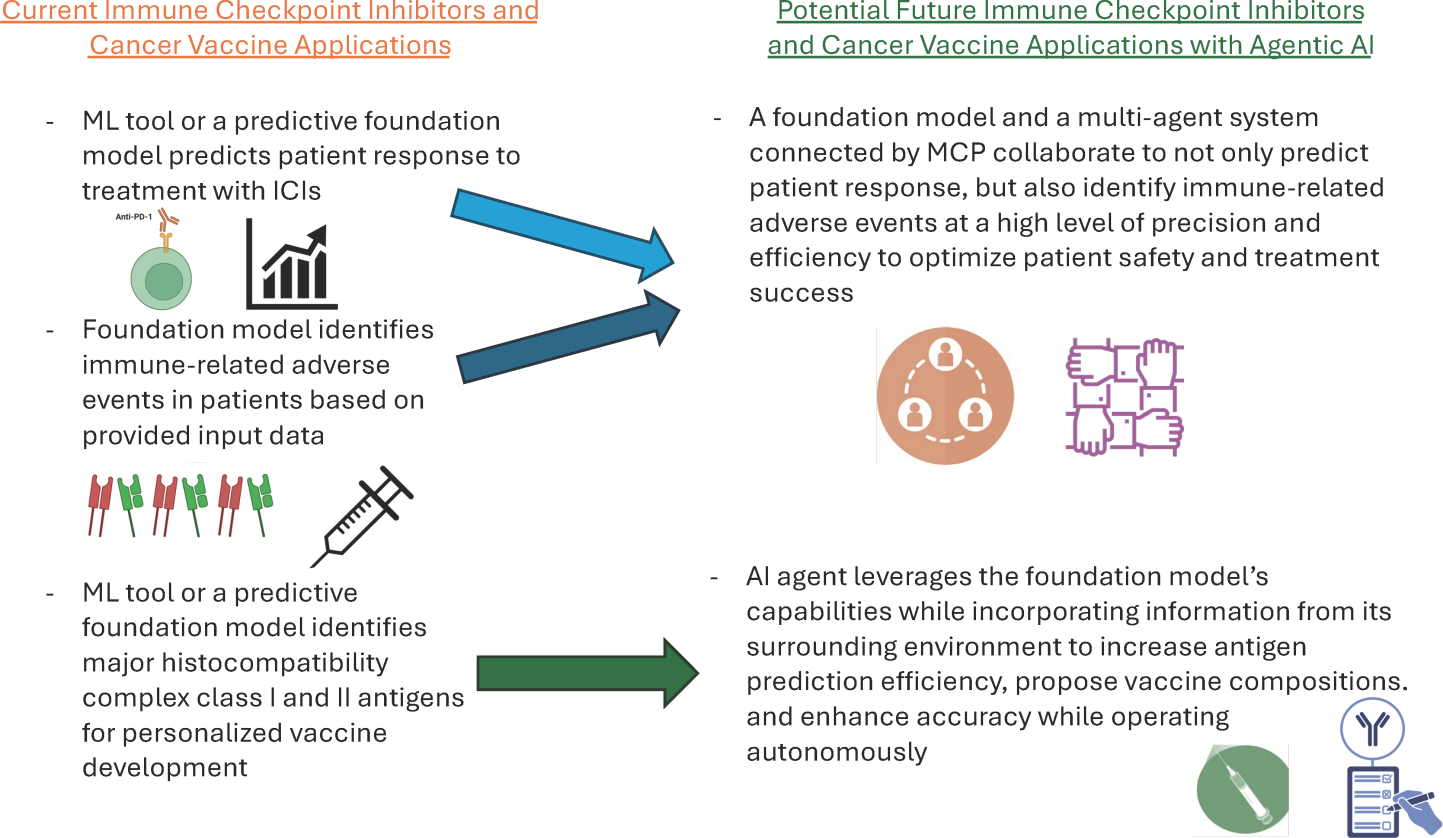
Current AI applications in immune checkpoint inhibitors and cancer vaccines, and how agentic AI can enhance these applications in the future. In ICIs, Current AI technologies are already showing promise for effectively predicting patient response to the treatment, as well as for identifying immune-related adverse events. AI agents, though, with the assistance of MCP interconnection, can further build upon these applications and carry them out in a collaborative manner that may enhance efficiency and treatment success. In cancer vaccines, agents can also leverage traditional AI capabilities to optimize vaccine development and delivery.

**Figure 3:**
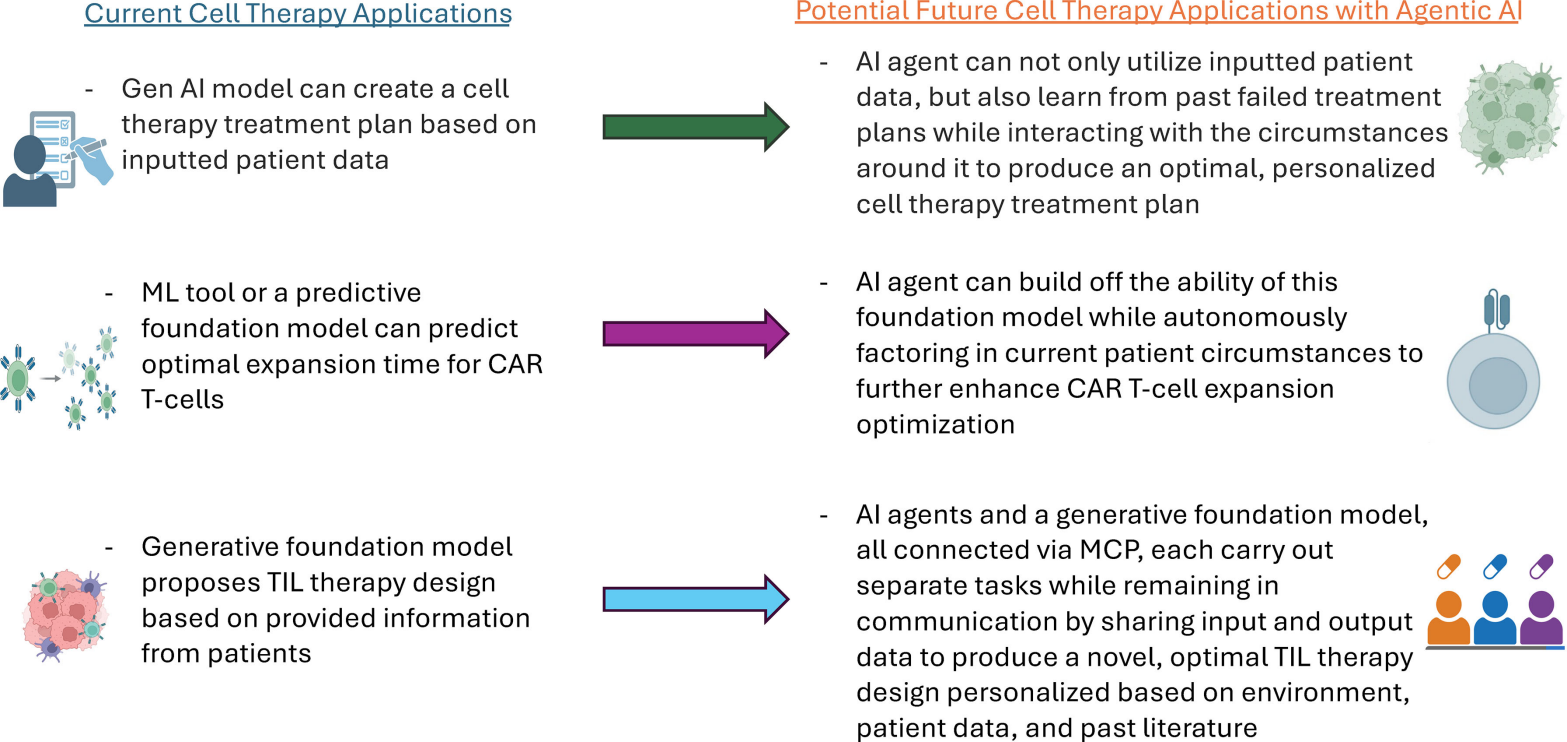
A contrast of current AI applications in cell therapy with future AI applications in the field with agentic AI. AI has already displayed the potential to strongly impact cell therapy. That said, agentic AI can build even further on already-innovative technology, such as foundation models, to additionally optimize cell therapy treatment. Utilizing the ability to learn from past errors and interact with the environment in an autonomous manner, agentic AI can increase efficiency and accuracy in a variety of cell therapy-related tasks, especially when connected via MCP.

**Figure 4:**
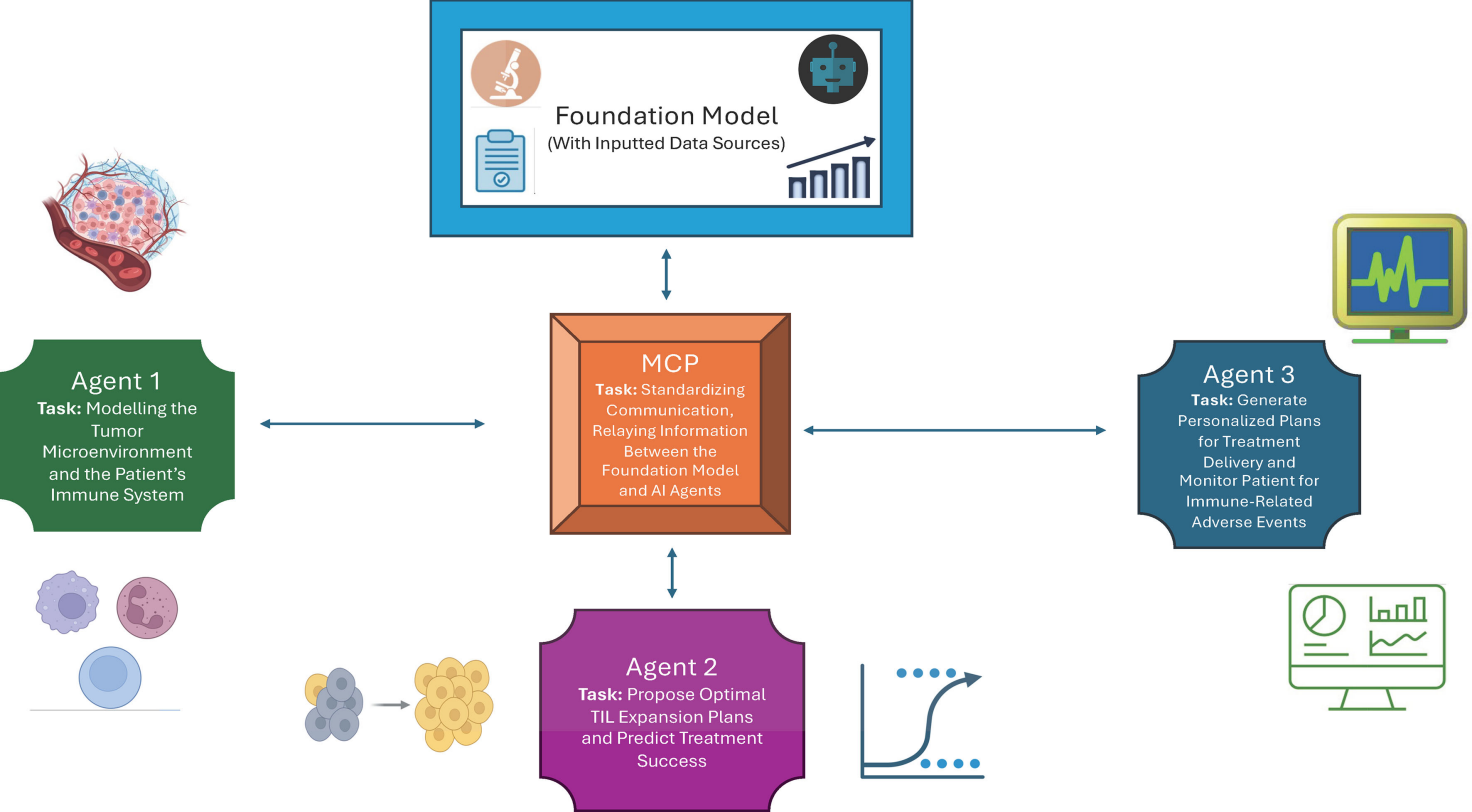
An example cell therapy application for a multi-agent system with a foundation model and MCP. In a multi-agent system, a foundation model can digest input data, with its capabilities leveraged by AI agents to perform distinct, valuable tasks in a cell therapy context. In the case of TIL therapy, individual agents can carry out modelling of the tumor microenvironment and the patient’s immune system, bringing forth TIL expansion protocols and predicting treatment success, or even treatment delivery plan generation and patient monitoring while treatment is in administration. All of these technologies are put in communication via MCP.

As discussed previously, AI agents and MCP can leverage and improve upon foundation models, potentially carrying out many tasks autonomously that technologies before it could not. **Figure 2** displays current AI applications in checkpoint inhibitors and vaccines, as an example, while depicting how AI agents and MCP can potentially build upon and enhance these applications in the future. With ICIs, individual agents can efficiently carry out tasks such as response prediction, treatment personalization based on patient information, adverse effect monitoring, and even novel drug discovery while exchanging information and results with each other as well as with foundation models through the MCP network. The scientific discovery aspect of AI agents is also quite fascinating. In fact, a so-called *AI co-scientist*, a multi-agent AI system, has been coined and developed to aid researchers in scientific discovery through complex tasks such as new hypothesis generation and experimental designs (59). In the future, tools like this one can be used to help discover novel immunotherapy drugs that improve and enhance treatment success and efficiency. Moreover, in cell therapies, AI agents can utilize information gathered from literature and patient data to collaboratively assist in the design of new CAR and TIL therapies, including the generation of constructs and antigen receptors, by analyzing sequences, learning from past studies carried out, and even interacting with various modes of patient information. **Figure 3** compares cell therapy applications that are possible with current forms of AI to potential future applications with agentic AI. For example, where foundation models such as scChat can monitor CAR T-cell exhaustion, a multi-agent system can leverage data sources digested by foundation models to oversee entire CAR T-cell therapy manufacturing processes. In this case, specialized agents could be responsible for modeling antigen-receptor interactions, generating genetic sequences for specific receptor interactions, predicting treatment outcomes, and performing other vital tasks. **Figure 4** depicts another specific cell therapy application that a multi-agent system can carry out with a foundation model and MCP. While there are various limitations surrounding agentic AI, If these issues are mitigated, foundation models, AI agents, and MCP can work effectively to bridge the gaps between optimal immunotherapy treatments and the current state of the field (59). These future applications underscore the potential of AI to raise the standard of effective cell therapies. However, in order to be used in such a manner, models must overcome various limitations, which will be discussed shortly (60).

### Limitations of AI in immunotherapy

Despite the transformative potential of AI, its widespread utilization is hindered by various limitations, including not only technical but also ethical concerns. For example, it is difficult for AI to replicate interpersonal discussions and conversations between doctors and patients. Patients want a personal relationship with physicians, so a human clinician must be regularly involved and present in all patient-related matters if AI is to be increasingly used as a clinical decision support tool (61). Human-AI interactions are complex and could be influenced by several competing factors (62), and arriving at the optimal decision needs to be a cooperative process. Moreover, physicians may not always be able to explain or verify AI outputs with the certainty required, in what is called “deskilling,” which can result from an overreliance on AI technologies (61). This can put patients in harm’s way, as misinformation can slip through the hands of physicians who may have few ways of knowing if an AI-based decision is beneficial or dangerous. Therefore, clinicians should ensure that they are not placing superfluous reliance on AI in a clinical setting (63). AI algorithms should also be consistently validated to prevent putting private patient data at risk via malfunctioning or hacked algorithms (64–67).

Despite the promise of general AI, validating its foundation models may be difficult unless the model is fine-tuned for a certain, defined task (68). In addition, the abnormal size of these models presents certain obstacles. For example, where a typical CNN architecture, which is specialized for image analysis, may have thousands or millions of parameters, which denote how much learning an AI model can carry out, foundation models usually contain billions and, more recently, trillions of parameters. This leads to increased computational needs that are met by training on a larger number of GPUs. As a result, the environmental concerns of AI are accentuated by heavily increased energy usage and carbon dioxide outputs (69). These limitations also may affect AI agents, which, as previously discussed, rely on foundation models for the processing of large amounts of data, as well as the feature extraction ability that foundation models have. Data quality is also an issue that must be addressed with AI agents; missing gaps or deficiencies in agentic datasets may prevent these technologies from attaining their full potential, and it can lead to superfluous results known as hallucinations that need to be guarded against.

### Limitations of the current study

As noted in our methods description, our study is not intended to be a comprehensive metanalysis of the literature rather than presenting the currents status of AI in immunotherapy through representative examples that covers immune checkpoint blockade treatment and identifying potent T-cells in the laboratory to develop effective cellular therapies. In terms of AI/ML technologies we primarily highlighted methods based on Gen AI and foundation models as the next frontier. Out of the possible 113 publications, only 42 were referenced and 13 were presented in detail as representative examples.

## Conclusions

Overall, AI has major potential for the enhancement and optimization of cancer immunotherapy. Both predictive and Gen AI have very important present and future roles, improved by the imminent transition from narrow to general AI. As these technologies become increasingly used together, immunotherapy can raise the medical standard of treatment success with novel therapy designs and the improvement of existing methods. With the integration of foundation models, agentic AI, and MCP, these technologies can reach their full potential at an expedited rate. While concerns surrounding AI in validation, cost, environmental effects, transparency, bias, and more are noteworthy, the potential of AI is still very promising. As limitations are overcome, AI has the potential to transform cancer immunotherapy and improve patient quality of life.
