# Supplementary figures and images for: Artificial intelligence for optimization of immunotherapy: current applications and transformative potential
Source: Front Immunol. 2026 May 29;17:1777580. doi: 10.3389/fimmu.2026.1777580 (PMC13260423; doi:10.3389/fimmu.2026.1777580)

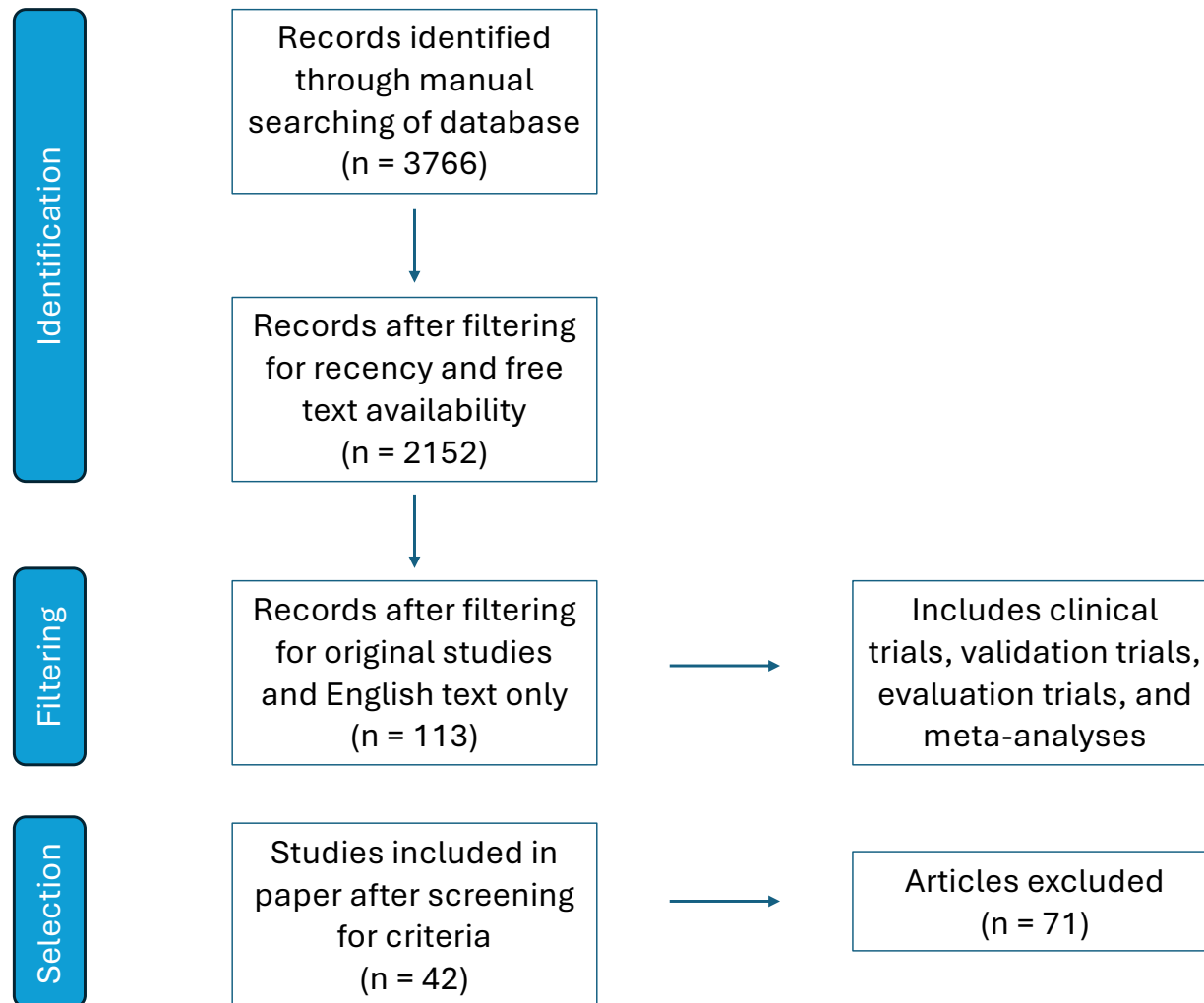

Supplement: Supplementary file 1 [file Image1.pdf]
