# Supplementary material for: Artificial intelligence for optimization of immunotherapy: current applications and transformative potential
Source: Front Immunol. 2026 May 29;17:1777580. doi: 10.3389/fimmu.2026.1777580 (PMC13260423; doi:10.3389/fimmu.2026.1777580)
Supplement: Supplementary file 2 [file Table1.pdf]

Table S1 Search Strategy_PubMed

	Search String	Number of studies found
#1	"Artificial intelligence" OR "Machine Learning" AND "CANCER"	65,445
#2	"Artificial intelligence" OR "Machine Learning" AND "Immunotherapy"	3,766
#3	#1 AND #2	2,954
#4	#1 AND #2 AND 5 Years AND Avallabile full text	2,152
#5	#1 AND #2 AND 5 Years AND Free full text AND Adaptive Clinical Trial AND Clinical Study AND Clinical Trial AND Clinical Trial, Phase I AND Clinical Trial, Phase II AND Clinical Trial, Phase III AND Clinical Trial, Phase IV AND Controlled Clinical Trial AND English Abstract AND Equivalence Trial AND Evaluation Study AND Multicenter Study AND Network Meta-Analysis AND Observational Study AND Practice Guideline AND Randomized Controlled Trial AND Technical Report AND Validation Study AND Humans	116
#6	English only	113
#7	Selection of References Describing Established AI Tools for Optimization of Immunotherapy (References 11-39, 41-53)	42
